# Extended Target Marginal Distribution Poisson Multi-Bernoulli Mixture Filter

**DOI:** 10.3390/s20185387

**Published:** 2020-09-20

**Authors:** Haocui Du, Weixin Xie

**Affiliations:** Automatic Target Recognition (ATR) Key Laboratory, Shenzhen University, Shenzhen 518060, China; hcdu@szu.edu.cn

**Keywords:** extended target tracking, gamma-Gaussian-inverse Wishart, Poisson multi-Bernoulli mixture

## Abstract

The existence of clutter, unknown measurement sources, unknown number of targets, and undetected probability are problems for multi-extended target tracking, to address these problems; this paper proposes a gamma-Gaussian-inverse Wishart (GGIW) implementation of a marginal distribution Poisson multi-Bernoulli mixture (MD-PMBM) filter. Unlike existing multiple extended target tracking filters, the GGIW-MD-PMBM filter computes the marginal distribution (MD) and the existence probability of each target, which can shorten the computing time while maintaining good tracking results. The simulation results confirm the validity and reliability of the GGIW-MD-PMBM filter.

## 1. Introduction

Multiple target tracking (MTT) involves estimating the state of an unknown number of targets in the presence of clutter [1,2,3]. The traditional MTT algorithm assumes each target is a point as one target generates at most one measurement in the sensor at each time step [3]. However, the rapid development of the modern high-resolution sensors makes the “point assumption” impractical because with such sensors, one target generates at least one measurement per time scan. The MTT problem with such sensors becomes an extended MTT problem [4,5]. Compared with the point target, the extended target can not only provide accurate target movement information, but also the target’ shape and size information, making it useful in the fields of robot recognition and positioning, moving crowd tracking, and tracking of close cars or large ships using the high-resolution sensors or automotive radars. Due to its wide application, the research on extended target tracking has received considerable attention from many scholars, making it a growing research hotspot.

As one of the most used extended target measurement models, The Poisson Point Process (PPP) model assumes a Poisson distributed random number of measurements are spatially distributed around the target at each time step [4,5]. To efficiently and correctly represent the spatially distributed measurements, many shape models have been developed, such as the random matrix model (RMM) [6,7,8,9,10,11,12,13,14,15], random hypersurface model (RHM) [16,17,18,19,20,21] and Gaussian process model (GPM) [22]. RMM assumes that the shape of the measurements can be approximated by an ellipse, and the measurements surrounding the centroid of the target obey a Gaussian distribution. RHM uses a more general star-convex shape to approximate the target contour. The GPM automatically learns the shape of the target through a Gaussian process and can give an analytic expression of its contour for an arbitrary shape target. Among the above three measurement models, the RMM has the smallest amount of calculation and is easy to implement, and can meet the requirements of many practical situations, so we use the RMM method in this paper.

Both in the point target-tracking filters and the extended target-tracking filters, the number of targets and the number of measurements are unknown and time-varying; thus, how to represent the targets and the measurements is a challenge. The random finite sets (RFS) theory [23], which represents the targets and the measurements as a finite variable set, is a suitable choice. To solve the point MTT problem, in the early stage, many RFS-based filters have been proposed, such as the Probability Hypothesis Density (PHD) filter [24,25,26], the Cardinalized Probability Hypothesis Density (CPHD) filter [27,28,29,30] and a series of multi-Bernoulli (MB) filters [31,32,33,34]. In recent years, scholars have proposed many RFS-based filters to solve the extended MTT problem, such as PHD for extended target tracking (ETT-PHD) [9,35,36,37], ETT-CPHD [38,39,40], gamma-Gaussian-inverse Wishart-Poisson multi-Bernoulli mixture (GGIW-PMBM) [40,41], GGIW implementation of the Labelled Multi-Bernoulli (GGIW-LMB) [42,43], and so on [22,44].

In the above-listed RFS-based filters, many kinds of conjugate priors are widely used, to give a closed-form solution of the posterior density. The prior conjugate refers to a time series of random finite set that satisfies the conjugate prior property; if the prediction is a Gaussian (Multi-Bernoulli) distribution, the update is also a Gaussian (Multi-Bernoulli) distribution. Using the conjugate prior, given enough parameters, the posterior distribution can be approximated arbitrarily.

In recent years, developing conjugate prior-based MTT filters has been a significant trend; the two kinds of most used conjugate priors in various MTT filters are the PMBM conjugate prior and the δ-Generalized Labelled Multi-Bernoulli (δ-GLMB) conjugate prior. In the δ-GLMB conjugate prior, the state of each target is added with a unique label, which is helpful to find each target’s trajectory. The PMBM prior conjugate divides the target set into two disjoint subsets, targets that have been detected and targets that have not yet been detected, and then processes the two subsets separately. This strategy can greatly reduce the target missed detection rate and improve tracking accuracy. In reference [40], it has been shown that the point target tracking filter based on PMBM conjugate prior outperforms the filter based on the GLMB conjugate prior in terms of tracking accuracy and computing time; thus, in this paper we used the PMBM conjugate prior.

In this paper, to solve the extended MTT problem and combine the PPP model and PMBM conjugate prior, we propose a GGIW implementation of a marginal distribution Poisson multi-Bernoulli mixture (MD-PMBM) filter on the basis of the GGIW-PMBM filter. The main difference between the GGIW-MD-PMBM filter and the GGIW-PMBM filter is that in the prediction step and the update step, instead of recursively propagating the joint state distribution of targets (GGIW-PMBM), the GGIW-MD-PMBM filter recursively propagates the marginal distribution and the existence probability of each target; such a strategy can effectively reduce the calculation cost while maintaining good tracking results.

The rest of the paper is organized as follows. Several model assumptions, such as the Bayesian model, PPP model, MBM model, motion model, measurement model, and PMBM conjugate prior, are discussed in Section 2. Section 3 presents the prediction step and the update step of the GGIW-MD-PMBM filter. Section 4 gives the four extended target filters’ experimental simulation results, which are summarized in Section 5.

## 2. Background

In this section, we outline various model assumptions, such as the Bayesian model [45,46,47], PPP model, Multi-Bernoulli (MB) process model, motion and measurement model, and PMBM conjugate prior model, which were used in the prediction and update steps of the GGIW-MD-PMBM filter.

### 2.1. Bayesian Model

We define the state of the *i*-th extended target at time step k as $\xi_{k}^{\left(i\right)}$, and the state of the target set at time step k can be written as

(1) $$X_{k}={\left\{\xi_{k}^{\left(i\right)}\right\}}_{i=1}^{N_{\xi ,k}},$$

where $N_{\xi ,k}=\;|X_{k}|$ is the number of the target, which is unknown and time-varying. The measurements (the union of target generated measurements and clutter) obtained from the sensor at time k are

(2) $$Z_{k}={\left\{z_{k}^{j}\right\}}_{j=1}^{N_{z,k}}$$

where $N_{z,k}=\;|Z_{k}|$ is the number of measurements. Let $Z^{k}$ denote the union of measurements set from time step 1 to *k*, that is, $Z^{k}=\;Z_{1}\cup Z_{2}\cup \cdots \cup Z_{k}$.

In many point target-tracking filters and extended target-tracking filters, the main purpose of using the Bayesian model is to recursively propagate the multi-target density distribution. In a Bayesian model, the multi-target set density, multi-target transition density, and the multi-target measurement likelihood are represented by $f_{k|k}(X_{k}|Z^{k})$,$\;f_{k|k-1}(X_{k}|X_{k-1})$, and$f_{k|k}(Z_{k}|X_{k})$, respectively. Using the Chapman–Kolmogorov equation, the multi-target set density can be defined as

(3) $$f_{k|k-1}(X_{k}|Z^{k-1})=\int f_{k|k-1}\left(X_{k}|X_{k-1}\right)f_{k-1|k-1}\left(X_{k-1}|Z^{k-1}\right)\delta X_{k-1},$$

and the Bayes update as

(4) $$f_{k|k}\left(X_{k}|Z^{k}\right)=\frac{f_{k|k}\left(Z_{k}|X_{k}\right)f_{k|k-1}\left(X_{k}|Z^{k-1}\right)}{\int f_{k|k}\left(Z_{k}|X_{k}\right)f_{k|k-1}\left(X_{k}|Z^{k-1}\right)\delta X_{k}},$$

### 2.2. PPP Model and Multi-Bernoulli (MB) Process Model

The PPP model, proposed by Gilholm et al. [4] and Granstrom et al. [5], is widely used in point target tracking filters and extended target tracking filters. In a PPP model, each target generates a Poisson distributed number of measurements, and each measurement is independent and identically distributed (i.i.d.). The Poisson rate μ and the spatial distribution f(·) determine the PPP intensity.

We define the spatial distribution of the target state $\xi_{k}^{\left(i\right)}$ as $f(\xi_{k}^{\left(i\right)})$. The Poisson density distribution of $X_{k}$ is

(5) $$f(X_{k})=e^{-\mu }\prod_{\xi_{k}^{\left(i\right)}\in X_{k}}\mu f(\xi_{k}^{\left(i\right)}).$$

The existence of a single target can be modeled by a Bernoulli RFS [14,31,32,33,34,40,41,42]. The cardinality of a Bernoulli RFS $X_{k}^{\left(i\right)}$ can either be 1 (with probability $r_{k}^{\left(i\right)}$) or empty (with probability $1-r_{k}^{\left(i\right)}$), and the Bernoulli density distribution of $X_{k}^{\left(i\right)}$ can be defined as

(6) $$f(X_{k}^{\left(i\right)})=\left\{ \begin{array}{ll} 1-r_{k}^{\left(i\right)}, & X_{k}^{\left(i\right)}=\phi \\ r_{k}^{\left(i\right)}f(\xi_{k}^{\left(i\right)}), & X_{k}^{\left(i\right)}=\{\xi_{k}^{\left(i\right)}\}\\ 0, & |X_{k}^{\left(i\right)}|\geq 2 \end{array}\right..$$

An MB RFS $X_{k}$ is the union of a limited number of independent Bernoulli RFS $X_{k}^{\left(i\right)}$, $X_{k}=\overset{N_{\xi ,k}}{\underset{i=1}{\cup }}X_{k}^{\left(i\right)}$. The MB density distribution of $X_{k}$ is

(7) $$f(X_{k})=(\overset{N_{\xi ,k}}{\underset{i=1}{∐}}\left(1-r_{k}^{\left(i\right)}\right))\sum_{1\leq i_{1}\neq \cdots \neq i_{n}\leq N_{\xi ,k}}\frac{r_{k}^{\left(i_{1}\right)}f(\xi_{k}^{\left(i_{1}\right)})}{1-r_{k}^{\left(i_{1}\right)}}\cdots \frac{r_{k}^{\left(i_{n}\right)}f(\xi_{k}^{\left(i_{n}\right)})}{1-r_{k}^{\left(i_{n}\right)}}.$$

In this paper, we divide the targets into the undetected target set and the detected target set. The PPP represents the distribution of undetected target measurements, and the MB mixture (MBM) represents the distribution of detected target measurements.

### 2.3. The Motion Model and Measurement Model

To simplify the calculation, at each time step, we assume each target evolves independently from the other targets, and the measurements generated by each target are independent of each other and independent of those generated by other targets.

We assume each birth target can be described by a PPP model, and its intensity is $D_{b}(x_{k})$. All the existing targets either survive with a probability $P_{D}(x_{k})$ or die with a probability $1-P_{D}(x_{k})$ from time step k to time step k+1. Each extended target state consists of three variables, the measurement rate $\gamma_{k}$, kinematic state $x_{k}$, and extent state $E_{k}$, that is, $\xi_{k}^{\left(i\right)}=\{\gamma_{k}^{\left(i\right)},x_{k}^{\left(i\right)},{Ε}_{k}^{\left(i\right)}\}$. Given $\gamma_{k}$, $x_{k}$ and $E_{k}$, the Markov motion model can be defined as

(8) $$\gamma_{k+1}=\gamma_{k},$$

(9) $$x_{k+1}=f(x_{k})+w_{k},$$

(10) $$E_{k+1}=M(x_{k})E_{k}M{\left(x_{k}\right)}^{T},$$

where $w_{k}$ is Gaussian process noise with zero mean and covariance Q, f(·) and M(·) are the transformation matrix of kinematic state and extent state, respectively.

The clutter measurements at time step k can be modeled as a PPP model with measurement rate λ  and spatial Poisson distribution c(z) . The clutter PPP density function is k(z)=λ c(z) . The measurements generated by each target at time step k are a Poisson distribution with a measurement rate $\gamma_{k}$. The measurement model is

(11) $$z_{k}=H_{k}x_{k}+\upsilon_{k},$$

where $H_{k}$ is the known observation model function, and $\upsilon_{k}$ is the Gaussian observation noise with zero mean and covariance $E_{k}$.

Assuming the measurements generated by a single target follow a Gaussian distribution, that is

(12) $$p(z_{k}|x_{k},\upsilon_{k})=N(z_{k};H_{k}x_{k},E_{k}).$$

The distribution of each target state can be expressed as

(13) $$\begin{array}{cc} p(\gamma_{k},x_{k},E_{k}|Z^{k}) & =p(\gamma_{k}|Z^{k})p(x_{k},E_{k}|Z^{k})p(E_{k}|Z^{k})\\ & =G(\gamma_{k};\alpha_{k|k},\beta_{k|k})N(x_{k};m_{k|k},P_{k|k}⊗E_{k})IW_{d}(E_{k};v_{k|k},V_{k|k})\;\\ & =GGIW(\xi_{k};\zeta_{k|k}) \end{array}$$

where $G(\gamma_{k};\alpha_{k|k},\beta_{k|k})$ is the Gamma distribution, $\alpha_{k|k}>0\;$ and $\beta_{k|k}>0$ are its shape parameter and inverse scale parameter; $N(x_{k};m_{k|k},P_{k|k}⊗E_{k})$ is the Gaussian distribution, $m_{k|k}$ and $P_{k|k}⊗E_{k}$ are its mean and covariance; $IW_{d}(E_{k};v_{k|k},V_{k|k})$ is the inverse Wishart distribution, $v_{k|k}$ and $V_{k|k}$ are its degrees of freedom and shape matrix. Note that $\zeta_{k|k}=\{\alpha_{k|k},\beta_{k|k},m_{k|k},P_{k|k},v_{k|k},V_{k|k}\}$ is a shorthand for the set of GGIW density parameters. The detailed implementation of GGIW prediction and update is given in [21,23,24,25,26].

### 2.4. PMBM Conjugate Prior

Existing studies [40,41,42] show that developing PMBM conjugate priors-based filters is a significant trend, due to their excellent tracking performance. In a PMBM model, the extended target set $X_{k}$ at time *k* is divided into two disjoint sets, undetected targets $X_{k}^{u}$ and detected targets $X_{k}^{d}$, then the PPP model is used to describe the distribution of undetected targets $X_{k}^{u}$, and the MBM model to describe the distribution of targets $X_{k}^{d}$ that have been detected at least once.

(14) $$X_{k}^{u},X_{k}^{d}:X_{k}=X_{k}^{u}\cup X_{k}^{d},X_{k}^{u}\cap X_{k}^{d}=\emptyset .$$

The PMBM set density can be defined as

(15) $$f_{k|k}(X_{k}|Z^{k})=\sum_{X_{k}^{u}\;⊎X_{k}^{d}=X_{k}\;}f_{{}_{k|k}}^{u}(X_{k}^{u}|Z^{k})\sum_{j\in J}\omega_{{}_{k|k}}^{d,j}f_{{}_{k|k}}^{d,j}(X_{k}^{d}|Z^{k}),$$

(16) $$f_{{}_{k|k}}^{u}(X_{k}^{u}|Z^{k})=e^{-\mu_{{}_{k|k}}^{u}}\prod_{\xi \in X_{k}^{u}}\mu_{{}_{k|k}}^{u}f_{{}_{k|k}}^{u}(\xi ),$$

(17) $$f_{{}_{k|k}}^{d,j}(X_{k}^{d}|Z^{k})=\sum_{\underset{i\in I^{j}}{⊎}X_{k}^{i}=X_{k}^{d}}\prod_{i\in I^{j}}f_{{}_{k|k}}^{d,j,i}(X_{k}^{i}),$$

where $f_{{}_{k|k}}^{d,j,i}(\cdot )$ is the Bernoulli set density. The MBM has  |J|  components, and J is the index set (or MBM component) of the MB in the MBM. $I^{j}$ is the Bernoulli index set of the *j-*th MB, that is, the *j-*th component has $\;|I^{j}|$ Bernoulli components. $\omega^{d,j}$ is the probability of the *j-*th MB component. The conjugacy property determines the intensities of the birth PPP and the initial undetected PPP, which are expressed by both GGIW mixture density.

Given the PMBM conjugate prior assumption, the extended target tracking is equivalent to recursively propagate the PMBM density parameters in the prediction step and the update step, which is shown in Section 3.

## 3. The GGIW-MD-PMBM Filter

In this section, the GGIW-MD-PMBM filter is presented. Instead of recursively propagating the joint state distribution of targets (GGIW-PMBM filter), the proposed GGIW-MD-PMBM filter recursively propagates the marginal distributions and the existence probabilities of each extended target. The GGIW-MD-PMBM filter can be modeled by the following assumptions:

(1) Clutter is uniformly distributed in the surveillance area with a given Poisson rate λ and is independent of targets’ distribution;

(2) Each extended target generates at least one measurement per time scan and evolves independently of the other targets;

(3) The birth PPP intensity is a GGIW mixture with a given Poisson rate $\mu^{b}$, weight $\omega^{b}$, and various density parameters $\varsigma^{b}$of GGIW;

(18) $$D_{k+1}^{b}=\mu_{k+1}^{b}\sum_{j=1}^{N_{k+1}^{b}}\omega_{k+1}^{\left(b,j\right)}GGIW(\xi_{k+1};\varsigma_{k+1}^{\left(b,j\right)}).$$

(4) The initial undetected PPP intensity is also a GGIW mixture with a known Poisson rate $\mu_{0}^{u}$, weight $\omega_{0}^{u}$, and various density parameters $\varsigma_{0}^{u}$ of GGIW.

(19) $$D_{0}^{u}=\mu_{0}^{u}\sum_{j=1}^{N_{0}^{u}}\omega_{0}^{\left(u,j\right)}GGIW(\xi_{0};\varsigma_{0}^{\left(u,j\right)}).$$

(5) The initial PMBM parameter is empty.

(20) $$\mu_{0|0}^{u}=0\;\mathrm{and}\;J_{0|0}=0.$$

We assume the birth PPP and the initial undetected targets are all GGIW mixture intensities; according to the PMBM conjugate property, each target density in the PMBM filter is also a GGIW density.

### 3.1. Prediction

#### 3.1.1. Detected Targets

According to the Chapman–Kolmogorov prediction (3) and the posterior PMBM density (15–16), the predicted distribution $f_{{}_{k|k-1}}^{d,j}(\xi_{k})$, $j=1,\cdots ,J_{d,k|k-1}$ and the existence probability $r_{{}_{k|k-1}}^{d,j}$ of each detected target at time step k are

(21) $$f_{{}_{k|k-1}}^{d,j}(\xi_{k})=GGIW(\xi_{k};\zeta_{{}_{k|k-1}}^{d,j}),j=1,\cdots ,J_{d,k|k-1},$$

(22) $$r_{{}_{k|k-1}}^{d,j}=p_{S}r_{{}_{k-1|k-1}}^{d,j},j=1,\cdots ,J_{d,k|k-1},$$

where $p_{S}$ is the survival probability, and $\zeta_{{}_{k|k-1}}^{d,j}$ are the predicted parameters. The weights and number of predicted MBM components are $\omega_{{}_{d,k|k-1}}^{j}=\omega_{d{,}_{k-1|k-1}}^{j}\;$ and$J_{d,k|k-1}=J_{d,k-1|k-1}\;$, respectively.

#### 3.1.2. Undetected Targets

To avoid missed detection, we regard previously undetected targets and current birth targets as the undetected target set, that is, the undetected targets at time step k include the undetected targets at time step k−1 and the birth targets at time step k (this paper does not consider the spawning targets). The predicted PPP for each undetected target at time step *k* is as follows:

(23) $$f_{{}_{k}}^{ub,j}(\xi_{k})\;=GGIW(\xi_{k};\zeta_{{}_{k}}^{ub,j}),j=1,\cdots ,N_{{}_{k}}^{ub},$$

(24) $$f_{{}_{k|k-1}}^{uu,j}(\xi_{k})\;=GGIW(\xi_{k};\zeta_{{}_{k|k-1}}^{uu,j}),j=1,\cdots ,N_{{}_{k|k-1}}^{uu}.$$

The existence probability is $\omega_{{}_{k}}^{ub,j}$ or $\omega_{{}_{k|k-1}}^{uu,j}$.

(25) $$\omega_{{}_{k}}^{ub,j},j=1,\cdots ,N_{{}_{k}}^{ub},$$

(26) $$\omega_{{}_{k|k-1}}^{uu,j}=\omega_{{}_{k-1|k-1}}^{uu,j}p_{S},j=1,\cdots ,N_{{}_{k|k-1}}^{uu},$$

where $N_{{}_{k}}^{ub}$ is the number of birth targets at time step k, and $N_{{}_{k|k-1}}^{uu}$ is the number of undetected targets at time step k−1. $\zeta_{{}_{k|k-1}}^{u,j}$ are the parameters ($\zeta_{{}_{k}}^{ub,j}$ or $\zeta_{{}_{k|k-1}}^{uu,j}$) of undetected targets at time step k. The predicted PPP for the undetected targets has a Poisson rate

(27) $$\mu_{{}_{k|k-1}}^{u}=\mu_{{}_{k}}^{ub}+P_{S}^{uu}\mu_{{}_{k-1|k-1}}^{uu},$$

where

(28) $$P_{S}^{uu}=\sum_{j=1}^{N_{{}_{k-1|k-1}}^{uu}}\omega_{{}_{k-1|k-1}}^{uu,j}p_{S}.$$

The existence probability of undetected targets $\omega_{{}_{k}}^{ub,j}$ and $\omega_{{}_{k|k-1}}^{uu,j}$ can be normalized, as $\omega_{{}_{k}}^{ub,j}=\frac{\mu_{{}_{k}}^{ub}}{\mu_{{}_{k|k-1}}^{u}}\omega_{{}_{k}}^{ub,j}$, $\omega_{{}_{k|k-1}}^{uu,j}=\frac{\mu_{{}_{k-1|k-1}}^{uu}}{\mu_{{}_{k|k-1}}^{u}}\omega_{{}_{k|k-1}}^{uu,j}$, and $\sum_{j=1}^{N_{{}_{k}}^{ub}}\omega_{{}_{k}}^{ub,j}+\;\sum_{j=1}^{N_{{}_{k}}^{uu}}\omega_{{}_{k|k-1}}^{uu,j}=1$. For simplicity, the existence probability of each undetected target at time k can be represented as $\omega_{{}_{k|k-1}}^{u,j}$, which includes $\omega_{{}_{k}}^{ub,j}$ and $\omega_{{}_{k|k-1}}^{uu,j}$.

Through the above analysis, in the prediction step, all extended target distributions and existence probabilities can be described as

(29) $$f_{k|k-1}^{j}(\xi_{k})\;,j=1,\cdots ,N_{k|k-1},$$

(30) $$\omega_{{}_{k|k-1}}^{j},j=1,\cdots ,N_{k|k-1},$$

where $N_{k|k-1}=J_{d,k|k-1}+N_{{}_{k|k-1}}^{uu}+N_{{}_{k}}^{ub}$. $f_{{}_{k|k-1}}^{j}(\xi_{k})=f_{{}_{k|k-1}}^{d,j}(\xi_{k})$ and $\omega_{{}_{k|k-1}}^{j}=r_{{}_{k|k-1}}^{d,j}$ for $1\leq j\leq J_{d,k|k-1}$, $f_{{}_{k|k-1}}^{j-J_{d,k|k-1}}(\xi_{k})=f_{{}_{k|k-1}}^{uu,j}(\xi_{k})\;$ and $\omega_{{}_{k|k-1}}^{j-J_{d,k|k-1}}=\omega_{{}_{k|k-1}}^{uu,j}$ for $J_{d,k|k-1}<j\leq J_{d,k|k-1}+N_{{}_{k|k-1}}^{uu}$, $f_{{}_{k|k-1}}^{j-J_{d,k|k-1}-N_{{}_{k}}^{uu}}(\xi_{k})=$$f_{{}_{k|k-1}}^{ub,j}(\xi_{k})\;$ and $\omega_{{}_{k|k-1}}^{j-J_{d,k|k-1}-N_{{}_{k}}^{uu}}=\omega_{{}_{k}}^{ub,j}$ for $j>J_{d,k|k-1}+N_{{}_{k|k-1}}^{uu}$.

### 3.2. Update

If each target predicted density is a PMBM at time k, according to the conjugate prior property, the updated density is also a PMBM. The updated MBM is given by the formulas (31) and (32), which contain the MB components predicted by each target in the previous processing step and their related data associations.

(31) $$f_{k|k}^{i}(\xi_{k}^{\left(i\right)}|Z^{k})=\sum_{Y\subseteq Z}\sum_{Ρ∠Y}\sum_{C_{C}\cap I^{j}\neq \emptyset \;}\omega_{k|k}^{i,Ρ}f_{{}_{k|k}}^{d,j}(\xi_{k}^{\left(i\right)}|Z^{k}),j=1,\cdots ,J_{d,k|k-1}+N_{{}_{k|k-1}}^{u},$$

(32) $$\omega_{k|k}^{i,Ρ}=\frac{\omega_{{}_{k|k-1}}^{j}L_{Ρ}L_{w}}{\sum_{Y\subseteq Z}\sum_{Ρ∠Y}\sum_{C_{C}\cap I^{j}\neq \emptyset \;}\omega_{k|k}^{i,Ρ}L_{Ρ}L_{w}},j=1,\cdots ,J_{d,k|k-1}+N_{{}_{k|k-1}}^{u}.$$

Here, the predicted likelihood $L_{Ρ}$ of partition and the predicted likelihood $L_{w}$ of each MB are as follows:

(33) $$L_{Ρ}=\prod_{\begin{array}{c} C_{C}\cap I^{j}=\emptyset \\ |C_{C}|>1 \end{array}}l_{C}\prod_{\begin{array}{c} C_{C}\cap I^{j}=\emptyset \\ |C_{C}|=1 \end{array}}\left(\kappa^{C_{C}}+l_{C}\right),$$

(34) $$L_{w}=\prod_{j}L_{C}.$$

In formulas (33) and (34), for each MB component in each partition cell, the data association can be divided into the following three types:

(1) The measurement set Z can be divided into the union of two disjoint sets, Z=Y∪(Z\Y), where Y corresponds to the clutter and the previously undetected targets’ measurements, and Z\Ycorresponds to the previously detected targets’ measurements.

(2) For set Y, form a partition of P of non-empty cells C; each cell contains clutter or target-generated measurements.

(3) For set Z\Y, the union of a set of index subsets $\;\{\;I^{j}\}$ can be used to represent the measurements related to the *j*-th MB component of the detected targets.

In the update step, the Bernoulli parameters of each detected target and the PPP parameters of each undetected target are determined, as given below.

#### 3.2.1. Detected Targets

In this paper, there are two types of detected targets at time k: The targets detected for the first time in the measurement set Y, and the previously detected targets are detected again in the measurement set Z\Y. The first detected targets are processed by the PPP model, and the second detected target is estimated with the existing MB. According to Equations (9) and (10), the spatial distribution $f_{{}_{k|k}}^{d,j}(\xi_{k})\;$ and existence probability $r_{{}_{k|k}}^{d,j}$ of each GGIW component of the detected target at time k are updated as follows:

(35) $$f_{{}_{k|k}}^{d,j}(\xi_{k}^{\left(j\right)})=\sum_{\underset{i\in I^{j}}{⊎}\xi_{k}^{i}=X_{k}^{d}}\prod_{i_{C}\cap I^{j}}f_{{}_{k|k}}^{d,j,i}(\xi_{k}^{\left(j\right)}),j=1,\cdots ,J_{d,k|k-1},$$

(36) $$r_{{}_{k|k}}^{d,j}=\frac{\omega_{{}_{k|k-1}}^{j}\prod_{C_{C}\cap I^{j}}r^{j,C}}{\sum_{j\in J}\omega_{{}_{k|k-1}}^{j}\prod_{i_{C}\cap I^{j}}r^{j,C}},j=1,\cdots ,J_{d,k|k-1}.$$

For each partition cell, its predicted likelihood $L_{C}$ is related to the predicted likelihood of each GGIW component in the spatial density, such as

(37) $$L_{C}=\left\{ \begin{array}{ll} \kappa^{C_{C}}+l_{C}, & if\;C\cap I^{j}=\emptyset \;,\;|C_{C}|=1\\ l_{C}, & if\;C\cap I^{j}=\emptyset \;,\;|C_{C}|>1\\ 1-r_{{}_{k|k-1}}^{d,j}p_{D}+r_{{}_{k|k-1}}^{d,j}p_{D}{\left(\frac{\beta_{{}_{k|k-1}}^{d,j,C}}{\beta_{{}_{k|k-1}}^{d,j,C}+1}\right)}^{\alpha_{{}_{k|k-1}}^{d,j,C}}, & if\;C\cap I^{j}\neq \emptyset \;,\;C_{C}=\emptyset \\ r_{{}_{k|k-1}}^{d,j}L_{k}^{u,j,C}, & if\;C\cap I^{j}\neq \emptyset \;,\;C_{C}\neq \emptyset \end{array}\right.$$

The predicted likelihood $L_{C}$ is determined by $C\cap I^{j}$ and the measurement cell$\;C_{C}$, as follows:

(1) When $C\cap I^{j}=\emptyset \;,\;|C_{C}|=1$, the predicted likelihood $L_{C}$ is an approximation of the predicted likelihood of the cell composed of the undetected target-generated measurements and the clutter measurements in the first detection process. Since this cell contains the clutter and undetected targets’ measurements, $L_{C}$ is related to $\kappa^{C_{C}}$ and $l_{C}$, where $\kappa^{C_{C}}$ is the predicted likelihood of clutter, and $l_{C}$ is the predicted likelihood of undetected target-generated measurements.

(2) When $C\cap I^{j}=\emptyset \;,\;|C_{C}|>1$, it means the targets detected for the first time are divided into multiple cells. If there are undetected targets, they must be contained in the above cells, thus $L_{C}$ is only related to $l_{C}$.

(3) When $C\cap I^{j}\neq \emptyset \;,\;C_{C}=\emptyset$, it means $L_{C}$ is the predicted likelihood when the *j*-th MB component of the previously detected target is an empty set.

(4) When $C\cap I^{j}\neq \emptyset \;,\;C_{C}\neq \emptyset$, the predicted likelihood $L_{C}$ is the approximation when the *j*-th MB component of the previously detected target is not empty. The calculation $L_{k}^{u,j,C}$ is presented in [39], Table 2.

The predicted likelihood $l_{C}$ of undetected targets can be defined as

(38) $$l_{C}=\mu_{{}_{k|k-1}}^{u}\sum_{j=1}^{N_{{}_{k|k-1}}^{u}}\omega_{{}_{k|k-1}}^{u,j}L_{k}^{u,j,C}.$$

According to the different values of $C\cap I^{j}$ and $\;C_{C}$, the likelihood probability $r^{j,C}$ can be defined as

(39) $$r^{j,C}=\left\{ \begin{array}{ll} \frac{l_{C}}{\kappa^{C_{C}}+l_{C}}, & if\;C\cap I^{j}=\emptyset \;,\;|C_{C}|=1\\ 1, & if\;C\cap I^{j}=\emptyset \;,\;|C_{C}|>1\\ \frac{r_{{}_{k|k-1}}^{d,j}q_{D}^{d,j,C}}{1-r_{{}_{k|k-1}}^{d,j}+r_{{}_{k|k-1}}^{d,j}q_{D}^{j,C}}, & if\;C\cap I^{j}\neq \emptyset \;,\;C_{C}=\emptyset \\ 1, & if\;C\cap I^{j}\neq \emptyset \;,\;C_{C}\neq \emptyset \end{array}\right..$$

The effective probability of the missed detection can be defined as

(40) $$q_{D}^{d,j,C}=1-p_{D}+p_{D}{\left(\frac{\beta_{{}_{k|k-1}}^{d,j,C}}{\beta_{{}_{k|k-1}}^{d,j,C}+1}\right)}^{\alpha_{{}_{k|k-1}}^{d,j,C}}.$$

The spatial distribution $f_{{}_{k|k}}^{d,j,i}(\xi_{k})$ of each measurement cell at time step k is as follows:

(41) $$f_{{}_{k|k}}^{d,j,C}(\xi_{k})=\left\{ \begin{array}{ll} \frac{\omega_{{}_{k|k-1}}^{u,j}L_{k}^{u,j,C}}{\sum_{j=1}^{N_{{}_{k|k-1}}^{u}}\omega_{{}_{k|k-1}}^{u,j}L_{k}^{u,j,C}}GGIW(\xi_{k};\zeta_{{}_{k|k}}^{d,j,C}), & if\;C_{C}\cap I^{j}=\emptyset \\ \frac{1-p_{D}}{q_{D}^{d,j,C}}GGIW(\xi_{k};\zeta_{{}_{k|k-1}}^{d,j,C})+\frac{p_{D}{\left(\frac{\beta_{{}_{k|k-1}}^{d,j,C}}{\beta_{{}_{k|k-1}}^{d,j,C}+1}\right)}^{\alpha_{{}_{k|k-1}}^{d,j,C}}}{q_{D}^{d,j,C}}\;G(\gamma_{k};\alpha_{{}_{k|k-1}}^{u,j,C},\beta_{{}_{k|k-1}}^{u,j,C}+1) & \\ \;\times N(x_{k};m_{{}_{k|k-1}}^{u,j,C},P_{{}_{k|k-1}}^{u,j,C})IW_{d}(X_{k};v_{{}_{k|k-1}}^{u,j,C},V_{{}_{k|k-1}}^{u,j,C}), & if\;C\cap I^{j}\neq \emptyset ,\;C_{C}=\emptyset \\ GGIW(\xi_{k};\zeta_{{}_{k|k}}^{d,j,C}), & if\;C\cap I^{j}\neq \emptyset ,\;C_{C}\neq \emptyset \end{array}\right..$$

The second equation in (41) uses a gamma-mixture reduction to reduce its bi-modal GGIW distribution to a uni-modal GGIW distribution [44]. Detailed calculations of parameter $\zeta_{{}_{k|k}}^{d,j,C}$ and prediction likelihood $L_{k}^{u,j,C}$ in the GGIW update are provided in [41].

#### 3.2.2. Undetected Targets

The detection probability $p_{D}$ is always less than 1, and the Poisson parameter $\gamma_{k}$ may be zero; these two cases may cause missed detection of targets. Therefore, we need to consider the above two situations, and the updated spatial density is

(42) $$\begin{array}{c} f_{{}_{k|k}}^{u,j}(\xi_{k})=\frac{(1-p_{D})\omega_{{}_{k|k-1}}^{u,j}}{\sum_{j=1}^{N_{{}_{k|k-1}}^{u}}q_{D}^{u,j}\omega_{{}_{k|k-1}}^{u,j}}GGIW(\xi_{k};\zeta_{{}_{k|k-1}}^{u,j})\;+\;\frac{p_{D}{\left(\frac{\beta_{{}_{k|k-1}}^{u,j}}{\beta_{{}_{k|k-1}}^{u,j}+1}\right)}^{\alpha_{{}_{k|k-1}}^{u,j}}\omega_{{}_{k|k-1}}^{u,j}}{\sum_{j=1}^{N_{{}_{k|k-1}}^{u}}q_{D}^{u,j}\omega_{{}_{k|k-1}}^{u,j}}G(\gamma_{k};\alpha_{{}_{k|k-1}}^{u,j},\beta_{{}_{k|k-1}}^{u,j}+1),\\ \times N(x_{k};m_{{}_{k|k-1}}^{u,j},P_{{}_{k|k-1}}^{u,j})IW_{d}(X_{k};v_{{}_{k|k-1}}^{u,j},V_{{}_{k|k-1}}^{u,j}),j=1,\cdots ,N_{{}_{k|k-1}}^{u} \end{array}$$

where $q_{D}^{u,j}$ is the effective probability of the undetected target, and $\mu_{{}_{k|k}}^{u}$ is the PPP Poisson rate of the undetected targets,

(43) $$q_{D}^{u,j}=1-p_{D}+p_{D}{\left(\frac{\beta_{{}_{k|k-1}}^{u,j}}{\beta_{{}_{k|k-1}}^{u,j}+1}\right)}^{\alpha_{{}_{k|k-1}}^{u,j}},$$

(44) $$\mu_{{}_{k|k}}^{u}=\mu_{{}_{k|k-1}}^{u}\sum_{j=1}^{N_{{}_{k|k-1}}^{u}}q_{D}^{u,j},$$

The existence probability is

(45) $$\omega_{{}_{k|k}}^{u,j}=\frac{(1-p_{D})\omega_{{}_{k|k-1}}^{u,j}}{\sum_{j=1}^{N_{{}_{k|k-1}}^{u}}q_{D}^{u,j}\omega_{{}_{k|k-1}}^{u,j}}+\frac{p_{D}{\left(\frac{\beta_{{}_{k|k-1}}^{u,j}}{\beta_{{}_{k|k-1}}^{u,j}+1}\right)}^{\alpha_{{}_{k|k-1}}^{u,j}}\omega_{{}_{k|k-1}}^{u,j}}{\sum_{j=1}^{N_{{}_{k|k-1}}^{u}}q_{D}^{u,j}\omega_{{}_{k|k-1}}^{u,j}},j=1,\cdots ,N_{{}_{k|k-1}}^{u}.$$

Note: Most of the mathematical details of formulas (42) and (45) are given in the Appendix A. The Gaussian and inverse Wishart parameters in formula (42) are the same in both cases, but the gamma parameters are different. Therefore, formula (42) can be reduced by gamma mixing [44] to a single-peak GGIW distribution and the number of GGIW components remains unchanged.

The main contribution of the GGIW-MD-PMBM proposed in this paper is to recursively propagate the marginal distribution and existence probability of each target through formulas (21), (22), (29), (34), (35), (42), and (45), which is different from the GGIW-PMBM in [40] that it recursively propagates the joint target state distribution and existence probability.

### 3.3. Complexity Reduction and Data Association

In the tracking process, as the number of targets increases, the number of unknown data associations, the number of components in the MBM, and the number of parameters in the PMBM greatly increase, which brings the problem of “combination explosion”. It is necessary to approximate the target density through reduction methods. Possible reduction methods include gating, clustering, pruning, merging, and recycling. In this paper, we focused on developing the approximate method to recursively propagate the marginal distribution of targets in the prediction and update steps. The complexity reduction and the data association process used in this paper follow the same methods used in [40], Section V; hence the complexity reduction and the data association are briefly discussed.

## 4. Simulation

In this simulation, we compared the proposed GGIW-MD-PMBM filter with the GGIW-PMBM, GGIW-LMB, and GGIW-PHD filters for multi-target trajectories in the four classic scenarios, which are provided in the excellent sample MATLAB code [40,41,42,43]. Each extended target can be defined as $x_{k}={\left[p_{k},v_{k}\right]}^{T}\in {\mathbb{R}}^{4}$, where $p_{k}\in {\mathbb{R}}^{2}$ is each target’s position and $v_{k}\in {\mathbb{R}}^{2}$ is the velocity. The motion model’s parameters used in Formulas (8)–(10) are

(46) $$f(x_{k})=\left[\begin{array}{cc} I_{2} & T_{s}I_{2}\\ 0 & I_{2} \end{array}\right]x_{k},\;Q=G\sigma_{a}^{2}I_{2}G^{T},\;G=\left[\begin{array}{l} \frac{T_{s}^{2}}{2}I_{2}\\ T_{s}I_{2} \end{array}\right],\;H_{k}=[I_{2}\;0_{2}],$$

where $\sigma_{a}$ is the acceleration standard deviation and $T_{s}=1s$ is the sampling time.

To evaluate the four algorithms’ performance, we used the computing time and the generalized optimal sub-pattern assignment (GOSPA) metric [47], the cutoff parameter c=10, and the ordered parameter p=1, among which, for the distance measurement, we use the Gaussian Wasserstein Distance metric [48]. We divided the GOSPA metric into three categories: localization error, false detection error, and miss detection error. For GGIW-LMB filter and GGIW-PHD filter, we extracted the target states by taking the mean vector of all Bernoullis whose existence probability is larger than 0.5. For the GGIW-PMBM filter and GGIW-MD-PMBM filter, target state extraction was performed similarly, but only from the MB component with the highest weight.

**Figure 1:**
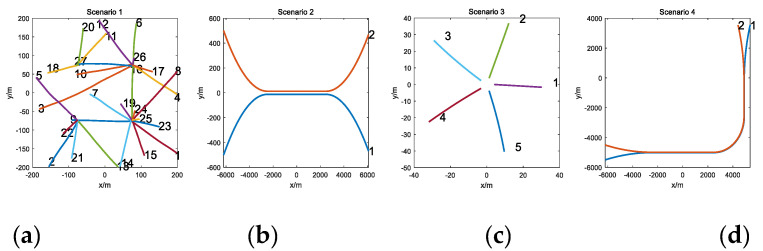
True target trajectories of four scenarios. (**a**) Twenty-seven targets, (**b**) separate/close/split, (**c**) dense birth, (**d**) nonlinear maneuver.

In the four scenarios, the target detection probability$p_{D}$, target survival probability $p_{S}$, the clutter Poisson rate λ, and the measurement Poisson rate γ used in different scenarios are given in Table 1. In the first scenario, there are 100 time steps, and 27 highly time-varying targets are randomly generated in four positions; this scenario aims to compare the four algorithms’ tracking performances with a high clutter density and high target number scenario. In the second scenario, there are 100 time steps, and two targets are born well separated, move close to each other, and then split; this scenario aims to compare the four algorithms’ tracking performance when highly close to each other. In the third scenario, there are 10 time steps, and five targets are born closely at the same time; this scenario aims to compare the four algorithms’ tracking performances of handling dense birth. In the fourth scenario, there are 300 time steps, and two targets first get close and then they maneuver closely before splitting; this scenario aims to compare the four algorithms’ tracking performance of seriously handling the data association problem. The target trajectories of different scenarios are given in Figure 1.

**Figure 2:**
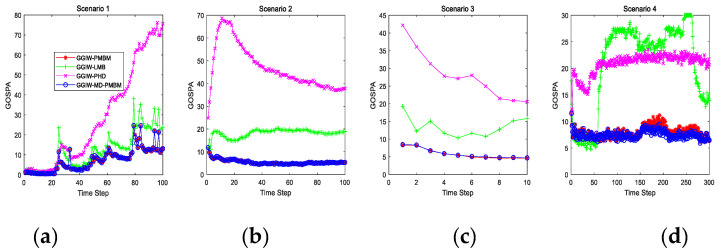
Comparison of the simulation results of the four algorithms of GOSPA. (**a**) Twenty-seven targets, (**b**) separate/close/split, (**c**) dense birth, (**d**) nonlinear maneuver.

Figure 2 gives the average GOSPA error of the four algorithms over 100 Monte Carlo (MC) trials. Table 2 shows the estimation errors and cycling time of the four algorithms in the four scenarios. From the simulation results in Figure 2 and Table 2, we can see that the proposed GGIW-MD-PMBM obtains the general minimum average GOSPA error, which outperforms the other three algorithms; GGIW-PMBM is the second, and GGIW-PHD obtains the highest average GOSPA error. As for the time consumption of each MC run, the GGIW-PHD has the lowest computational cost, GGIW-LMB is the second, and GGIW-MD-PMBM is the third. Compared with other results of normalized location error, the number of missed targets, and the number of false detection, the proposed GGIW-MD-PMBM algorithm is generally better than the other three algorithms. In the third scenario, the GGIW-PMBM filter outperformed the GGIW-MD-PMBM filter in terms of the location error and the number of false detections; this is because the targets in this scenario are intensively generated at the same location and at the same time. Compared with the joint state distribution (GGIW-PMBM), the marginal distribution (GGIW-MD-PMBM) has a larger deviation, but as time increases, the targets gradually move away, and the average GOSPA error of the GGIW-MD-PMBM filter is still lower than the GGIW-PMBM filter. The tracking performance of each algorithm in the fourth scenario is worse than in the other three scenarios; this is because the data association in the fourth scenario is worse than in the other three scenarios.

## 5. Conclusions

In this paper, we propose an efficient filter to solve the extended target tracking problem. Unlike the existing GGIW-PMBM filter that recursively propagates the joint state distribution of targets, the proposed GGIW-MD-PMBM filter recursively propagates the marginal distributions and the existence probabilities of each extended target. Comparing the other three extended target filters in four classic scenarios, the experimental results show that the proposed filter in this paper improves tracking accuracy and reduces computing time.

## Figures and Tables

**Table 1:** The parameters used in the four scenarios.

Scenario	$p_{D}$	$p_{S}$	λ	γ
Scenario 1	0.90	0.99	60	{7,8,9}
Scenario 2	0.98	0.99	10	{10,20}
Scenario 3	0.90	0.99	20	10
Scenario 4	0.98	0.99	10	{10,20}

**Table 2:** Simulation results: the sum of estimation errors and cycling time; GO-GOSPA; LE-normalized location error; NM-number of missed targets; NF-number of false detection; CT-cycling time.

Scenarios		GGIW-PMBM	GGIW-LMB	GGIW-PHD	GGIW-MD-PMBM
Scenario 1	GO	732.8	1246.7	2873.2	729
	LE	56.5	76.6	60.7	56.3
	NM	61.3	481.0	2311.6	60.7
	NF	141.4	96.2	108.5	137
	CT	62.5	8.0	4.2	46.2
Scenario 2	GO	550.7	1818.2	4699.5	550.5
	LE	268.0	133.9	562.3	265.1
	NM	5.6	1479.5	997.5	5.0
	NF	16.9	193.8	3083.2	10.8
	CT	18.6	7.0	0.3	11.4
Scenario 3	GO	59.2	134.8	280.4	58.3
	LE	9.3	11.5	23.1	9.5
	NM	11.2	73.8	174.7	10.6
	NF	2.1	12.1	31.8	2.5
	CT	1.2	0.4	0.1	1.1
Scenario 4	GO	2835.6	6266.3	6257.8	2236.7
	LE	1011.2	869.3	176.8	1002.8
	NM	253.6	3770.6	5601.2	139.4
	NF	175.0	1445.2	470.8	106.2
	CT	49.7	6.5	2.2	40.6

## Appendix A

Let $p_{k,D}^{e}$ be the effective probability of target detection [35] (the set of target detections is nonempty with probability $1-e^{-\gamma (j)}$), then the updated PPP intensity corresponding to previously existing targets that are not detected is

(A1) $$\begin{array}{cc} D_{{}_{k|k}}^{u}(\xi_{k}) & =\sum_{j=1}^{N_{{}_{k|k-1}}^{u}}\left(1-p_{k,D}^{e}\right)\omega_{{}_{k|k}}^{u,j}GGIW(\xi_{k};\zeta_{{}_{k|k-1}}^{u,j})\\ & =\sum_{j=1}^{N_{{}_{k|k-1}}^{u}}p^{\left(j\right)}(\gamma_{k})N(x_{k};m_{{}_{k|k-1}}^{u,j},P_{{}_{k|k-1}}^{u,j})IW_{d}(X_{k};v_{{}_{k|k-1}}^{u,j},V_{{}_{k|k-1}}^{u,j}) \end{array}$$

where $p^{\left(j\right)}(\gamma_{k})$ is

(A2) $$p^{\left(j\right)}(\gamma_{k})=(1-p_{D})\omega_{{}_{k|k-1}}^{u,j}G(\gamma_{k};\alpha_{{}_{k|k-1}}^{u,j},\beta_{{}_{k|k-1}}^{u,j})+p_{D}{\left(\frac{\beta_{{}_{k|k-1}}^{u,j}}{\beta_{{}_{k|k-1}}^{u,j}+1}\right)}^{\alpha_{{}_{k|k-1}}^{u,j}}\omega_{{}_{k|k-1}}^{u,j}G(\gamma_{k};\alpha_{{}_{k|k-1}}^{u,j},\beta_{{}_{k|k-1}}^{u,j}+1).$$

At this time, all existence probabilities of the undetected targets can be normalized, and the existence probability and spatial density of each target can be as follows:

(A3) $$\omega_{{}_{k|k}}^{u,j}=\frac{(1-p_{D})\omega_{{}_{k|k-1}}^{u,j}}{\sum_{j=1}^{N_{{}_{k|k-1}}^{u}}q_{D}^{u,j}\omega_{{}_{k|k-1}}^{u,j}}+\frac{p_{D}{\left(\frac{\beta_{{}_{k|k-1}}^{u,j}}{\beta_{{}_{k|k-1}}^{u,j}+1}\right)}^{\alpha_{{}_{k|k-1}}^{u,j}}\omega_{{}_{k|k-1}}^{u,j}}{\sum_{j=1}^{N_{{}_{k|k-1}}^{u}}q_{D}^{u,j}\omega_{{}_{k|k-1}}^{u,j}},j=1,\cdots ,N_{{}_{k|k-1}}^{u}$$

(A4) $$f_{{}_{k|k}}^{u,j}(\xi_{k})=f_{{}_{1,k|k}}^{u,j}(\xi_{k})+f_{{}_{2,k|k}}^{u,j}(\xi_{k}),j=1,\cdots ,N_{{}_{k|k-1}}^{u}$$

where $f_{{}_{1,k|k}}^{u,j}(\xi_{k})$ represents the spatial density in the case of a missed detection caused by $p_{D}<1$, and $f_{{}_{2,k|k}}^{u,j}(\xi_{k})$ represents the spatial density in the case of a missed detection caused by $\gamma_{k}=0$.

(A5) $$f_{{}_{1,k|k}}^{u,j}(\xi_{k})=\frac{(1-p_{D})\omega_{{}_{k|k-1}}^{u,j}}{\sum_{j=1}^{N_{{}_{k|k-1}}^{u}}q_{D}^{u,j}\omega_{{}_{k|k-1}}^{u,j}}GGIW(\xi_{k};\zeta_{{}_{k|k-1}}^{u,j})\;$$

(A6) $$f_{{}_{2,k|k}}^{u,j}(\xi_{k})=\frac{p_{D}{\left(\frac{\beta_{{}_{k|k-1}}^{u,j}}{\beta_{{}_{k|k-1}}^{u,j}+1}\right)}^{\alpha_{{}_{k|k-1}}^{u,j}}\omega_{{}_{k|k-1}}^{u,j}}{\sum_{j=1}^{N_{{}_{k|k-1}}^{u}}q_{D}^{u,j}\omega_{{}_{k|k-1}}^{u,j}}G(\gamma_{k};\alpha_{{}_{k|k-1}}^{u,j},\beta_{{}_{k|k-1}}^{u,j}+1)N(x_{k};m_{{}_{k|k-1}}^{u,j},P_{{}_{k|k-1}}^{u,j})IW_{d}(X_{k};v_{{}_{k|k-1}}^{u,j},V_{{}_{k|k-1}}^{u,j})$$
